# Physical exercise regulates microglia in health and disease

**DOI:** 10.3389/fnins.2024.1420322

**Published:** 2024-06-07

**Authors:** Alexandra O. Strohm, Ania K. Majewska

**Affiliations:** ^1^Department of Environmental Medicine, University of Rochester Medical Center, Rochester, NY, United States; ^2^Department of Neuroscience, University of Rochester Medical Center, Rochester, NY, United States; ^3^Del Monte Institute for Neuroscience, University of Rochester Medical Center, Rochester, NY, United States; ^4^Center for Visual Science, University of Rochester Medical Center, Rochester, NY, United States

**Keywords:** microglia, physical exercise, neurodevelopment, neurodegeneration, aging, environmental exposure

## Abstract

There is a well-established link between physical activity and brain health. As such, the effectiveness of physical exercise as a therapeutic strategy has been explored in a variety of neurological contexts. To determine the extent to which physical exercise could be most beneficial under different circumstances, studies are needed to uncover the underlying mechanisms behind the benefits of physical activity. Interest has grown in understanding how physical activity can regulate microglia, the resident immune cells of the central nervous system. Microglia are key mediators of neuroinflammatory processes and play a role in maintaining brain homeostasis in healthy and pathological settings. Here, we explore the evidence suggesting that physical activity has the potential to regulate microglia activity in various animal models. We emphasize key areas where future research could contribute to uncovering the therapeutic benefits of engaging in physical exercise.

## Introduction

It is largely accepted that physical exercise (PE) can promote brain health and cognitive function. Reports in humans show that moderate to vigorous PE can enhance cognition (Colcombe and Kramer, 2003; Angevaren et al., 2008; Hamer et al., 2018; Cheval et al., 2023; James et al., 2023; Li W. et al., 2023). However, the cellular mechanisms that underlie this phenomenon are still an active area of exploration. Traditionally, studies have examined how PE regulates wiring of neuronal connections to enhance cognitive function (Festa et al., 2023). However, recent focus has shifted toward how exercise may regulate inflammation and the immune response in the central nervous system (CNS).

Microglia are the resident immune cells of the CNS responsible for mediating inflammatory responses, tissue maintenance, and synapse remodeling (Li and Barres, 2018; Whitelaw et al., 2023). Many therapeutics are designed to target microglia activity, as it is tightly linked to neuronal health and cognitive function. Under homeostatic conditions, microglia are highly ramified and maintain discrete territories with uniform dispersal throughout the brain (Nimmerjahn et al., 2005). Plasticity, the brain’s ability to adapt both functionally and structurally to intrinsic and extrinsic stimuli, is an ongoing process that begins in development and continues throughout a lifespan. Microglia are active participants in plasticity, perpetually undergoing functional and structural changes, extending and retracting their processes to survey their environment and monitor the functional state of the brain (Nimmerjahn et al., 2005; Wake et al., 2009). In doing this, microglia dynamically interact with synaptic elements to facilitate synapse remodeling (Wake et al., 2009; Tremblay et al., 2010; Paolicelli et al., 2011; Nebeling et al., 2023). These interactions are governed by a variety of signaling pathways and molecules reviewed in Whitelaw et al. (2023), including norepinephrine and BDNF which are known to be produced during PE (see “Exercise Increases Known Modulators of Microglia Activity”). Beyond synapse regulation, microglia serve critical functions in regulating myelination, injury and inflammatory responses, and neurogenesis, reviewed in Bobotis et al. (2024). Perturbations of microglial function have been described in numerous neurological diseases and disorders. Functional changes in microglia are often accompanied by alterations in microglial morphology, number, distribution, and phenotype characterized by altered expression of various molecules (Paolicelli et al., 2022; Bobotis et al., 2024). In certain circumstances, microglial activity can decrease or shift to different functions resulting in a diminished ability to migrate, respond to injury and clear debris (Hefendehl et al., 2014; Thomas et al., 2022). In other cases, microglia can engage in excessive synaptic pruning, or release pro-inflammatory factors that can contribute to cognitive decline (Hong et al., 2016; Pinto et al., 2020). By targeting microglia activity, these processes can be differentially impacted in health and disease. Thus, discovering strategies to modulate microglia activity is of great interest. However, the overall effects of PE on deficits in function and cognition depend on the timing of the exercise intervention. For more discussion on how changes in microglial activity and function can impact the brain during development as well as in health and disease, please see Paolicelli and Ferretti (2017) and Gao et al. (2023).

**FIGURE 1:**
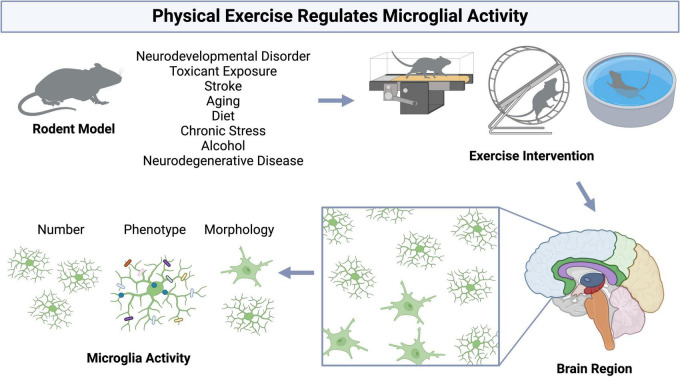
Physical exercise regulates microglia activity in rodent models. Figure made with Biorender.com.

**FIGURE 2:**
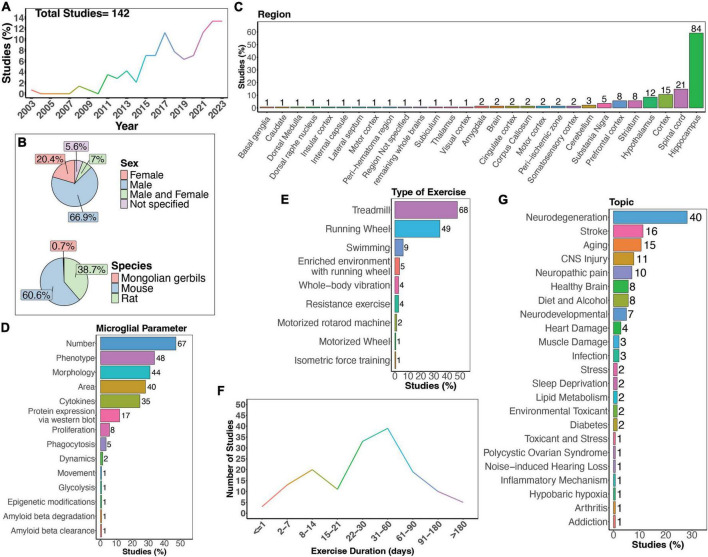
Summary of physical exercise and microglia activity. **(A)** Percent of studies published each year within the scope of this review. Total number of studies included in review = 142. **(B)** Percent of studies utilizing male, female, or both sexes (top) and species used (bottom). **(C)** Percent of studies which examine microglia in the brain regions shown. **(D)** Percent of studies which measured microglia parameters shown. **(E)** Percent of studies utilizing different types of exercise paradigms. **(F)** Number of studies implementing exercise paradigms of various durations. **(G)** Percent of studies published on each topic shown. Numbers of studies are included next to the bars for percentage plots. For **(C–E)**, percentages exceed 100% as some studies measured more than one microglial parameter, examined more than one brain region, or implemented more than one form of exercise.

This review focuses on how PE has been shown to modulate microglia function in different animal models and highlight areas where further research could be beneficial (Figure 1). An overview of the comprehensive review process is shown in Supplementary Figure 1. Studies were reviewed from the PUBMED search query: ((((exercise[Title/Abstract]) OR (physical exercise[Title/Abstract])) OR (physical activity[Title/Abstract])) AND (microglia[Title/Abstract])) NOT (review[Publication Type]). This resulted in a list of studies which were published over a twenty-year span (between 2003 and November 21, 2023; Figure 2A). Select research studies within the scope of this review which were found manually outside the search parameters stated above were also included. Only peer reviewed, primary research studies were included. Data was manually extracted from each study on microglia parameters in various exercise animal models. Studies which only analyzed cytokines were excluded due to their possible contributions from multiple cell types. For a detailed review on how different exercise paradigms impact pro and anti-inflammatory cytokines (please see Mee-Inta et al., 2019). Cytokines were included as a parameter measured if the study measured cytokines from isolated hippocampal microglial or in conjunction with other microglial parameters. Phenotypic parameters encompass measurements of microglia expression of different markers and molecules, including, but not limited to cluster of differentiation 68 (CD68), C-X3-C motif chemokine receptor 1 (CX3CR1), cluster of differentiation 86 (CD86), major histocompatibility complex class 2 (MHCII), insulin-like growth factor 1 (IGF-1), brain-derived neurotrophic factor (BDNF), Complement C1q A Chain (C1QA), mannose receptor (CD206), and galectin-3 (Gal-3). Studies were excluded if they were not available in English, did not use animal models, did not directly measure microglia parameters, or did not include an exercise intervention (Supplementary Figure 1). Studies using the types of exercise described in Figure 2E were included in this review, which led to the exclusion of one study using “foraging exercise.” All studies included in this review and their information is reported in Supplementary Table 1.

## Physical exercise and microglial activity

There is a clear increase in interest in the effects of physical exercise on microglial function, with more studies being published over time which examine microglial functions in response to physical exercise (Figure 2A). Of these studies, the majority used male mice (66.9%), and fewer used females (20.4%). A small percent of studies examined both males and females (7%; Figure 2B). Studies in humans have demonstrated sex-differences in sensitivity to exercise, with women showing smaller BDNF changes after exercise on average (Szuhany et al., 2015), highlighting the necessity to use both sexes in animal studies. Furthermore, as microglia phenotypes are sex-dependent (Guneykaya et al., 2018; Ochocka and Kaminska, 2021), there is a clear need for more studies which directly compare male and female responses to physical exercise. Most studies used mouse models (Figure 2B), and while many different brain regions were examined, the hippocampus was the most frequently studied brain area (Figure 2C). As microglia are regionally heterogenous and exhibit functional differences in different regions (Tan et al., 2020; Ochocka and Kaminska, 2021), it is important for studies to perform regional comparisons in the future. Various microglial parameters were assessed, with most studies examining cell number, phenotype, and morphology (Figure 2D). Surprisingly, few have explored how exercise may influence microglia dynamic activities, such as process motility and surveying capacity or soma translocation (Figure 2D). When examining the different types of exercise implemented, the majority used treadmill running, a running wheel, and swimming (Figure 2E). Additionally, different durations of physical exercise were used, where most animals underwent 1 to 2 months of exercise (Figure 2F). Lastly, most studies examined the effects of physical exercise in models of neurodegeneration, followed by stroke, and aging (Figure 2G).

## Physical exercise and microglia in the healthy brain

**FIGURE 3:**
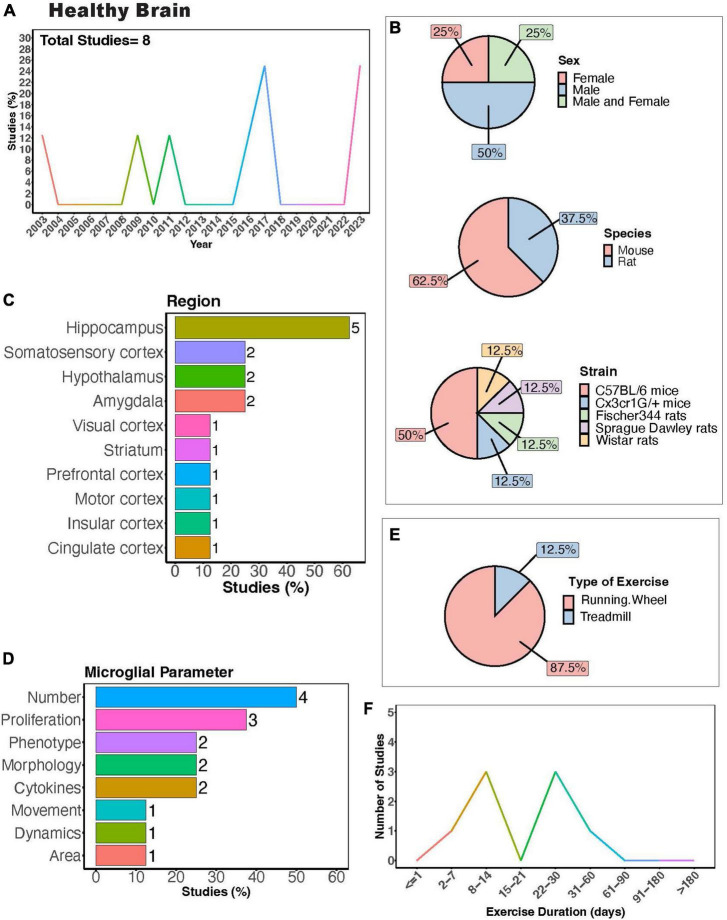
Physical exercise and microglia in the healthy brain. **(A)** Percent of studies published each year examining effects of physical exercise on microglia in a healthy setting. Total number of studies = 8. **(B)** Percent of studies utilizing male, female, or both sexes (top), various species (middle), and strain (bottom). **(C)** Percent of studies which examine microglia in the brain regions shown. **(D)** Percent of studies which measured microglia parameters shown. **(E)** Percent of studies utilizing running wheel or treadmill exercise. **(F)** Number of studies implementing exercise paradigms of various durations. Numbers of studies are included next to the bars for percentage plots. For **(C,D)**, percentages exceed 100% as some studies measured more than one microglial parameter or examined more than one brain region.

In the healthy brain, exercise can improve cognitive function in both humans (Colcombe and Kramer, 2003; Angevaren et al., 2008; Hamer et al., 2018; Cheval et al., 2023; James et al., 2023) and rodents (van Praag et al., 1999a,b). Understanding how exercise modulates microglia activity in the healthy brain could provide insight into the mechanisms behind the positive benefits of exercise, as microglia play important roles in circuit maturation and synaptic remodeling in different brain areas (Wake et al., 2009; Tremblay et al., 2010; Paolicelli et al., 2011; Nebeling et al., 2023). There are a limited number of investigations into how exercise may regulate microglia activity exclusively under healthy conditions using rodent models (Figure 3A). Males were most frequently used in experiments, with half as many studies employing both males and females (Figure 3B). Mouse models were more commonly employed compared to rat models, with C57BL/6 mice being the most utilized strain (Figure 3B). Most studies examined the hippocampus (<60%, Figure 3C), highlighting a gap in studies examining exercise regulation of microglia in a healthy setting in other brain areas, such as the cerebellum. Studies measured microglial parameters including number, proliferation, phenotype, morphology, cytokines, dynamics, and area (Figure 3D). Most used a running wheel for exercise (Figure 3E) and animals underwent exercise for either 1-2 or 3-4 weeks (Figure 3F). In healthy rats and mice, increased microglial numbers have been reported in the hippocampus (Xu et al., 2016; Sun et al., 2017) and hypothalamus (Soch et al., 2016). In healthy mice, 10 days of voluntary wheel running (VWR) can change microglial proliferation within specific brain areas, with increased proliferation reported in several cortical layers and the hippocampus (Ehninger and Kempermann, 2003; Olah et al., 2009; Ehninger et al., 2011). Despite this increase in proliferation, no changes in morphology were observed (Olah et al., 2009). However, longer durations of VWR can induce changes in the hippocampal microglial phenotype (alterations in CD86/MHCII+, mammalian target of rapamycin (mTOR), CX3CR1 expression) in a healthy setting (Kohman et al., 2013; Lloyd et al., 2017; Williams et al., 2023). Until recently, the effects of exercise on the normal basal surveillance carried out by microglial processes had not been examined. In recent a study, we found one month of VWR did not have effects on primary somatosensory cortical (S1) microglial number, morphology, or dynamics in healthy male or female mice (Strohm et al., 2024). However, it is possible that other forms of exercise, such as treadmill running, or longer durations of exercise could impact microglial dynamics. It is also possible that microglial dynamics are more sensitive to exercise in other brain areas, such as the hippocampus. Of note, hippocampal microglial process dynamics can be regulated by BNDF (Onodera et al., 2021), which is increased with exercise (see below). This was demonstrated by Onodera et al. who observed increases in hippocampal microglial process motility and engulfment of mossy fibers when BDNF was pharmacologically blocked in hippocampal slices (Onodera et al., 2021). Whether exercise is sufficient to alter microglial dynamics in the hippocampus through changes in BDNF remain to be determined. A comprehensive study of different PE paradigms may provide insight into the regional and sex- dependent effects of exercise microglia in healthy settings. Hence, further research is required to draw conclusions regarding the capacity of physical exercise to regulate microglia activity under healthy conditions and the consequences of exercise-induced microglial changes on circuit maintenance.

## Physical exercise and microglia in neurodevelopmental models

**FIGURE 4:**
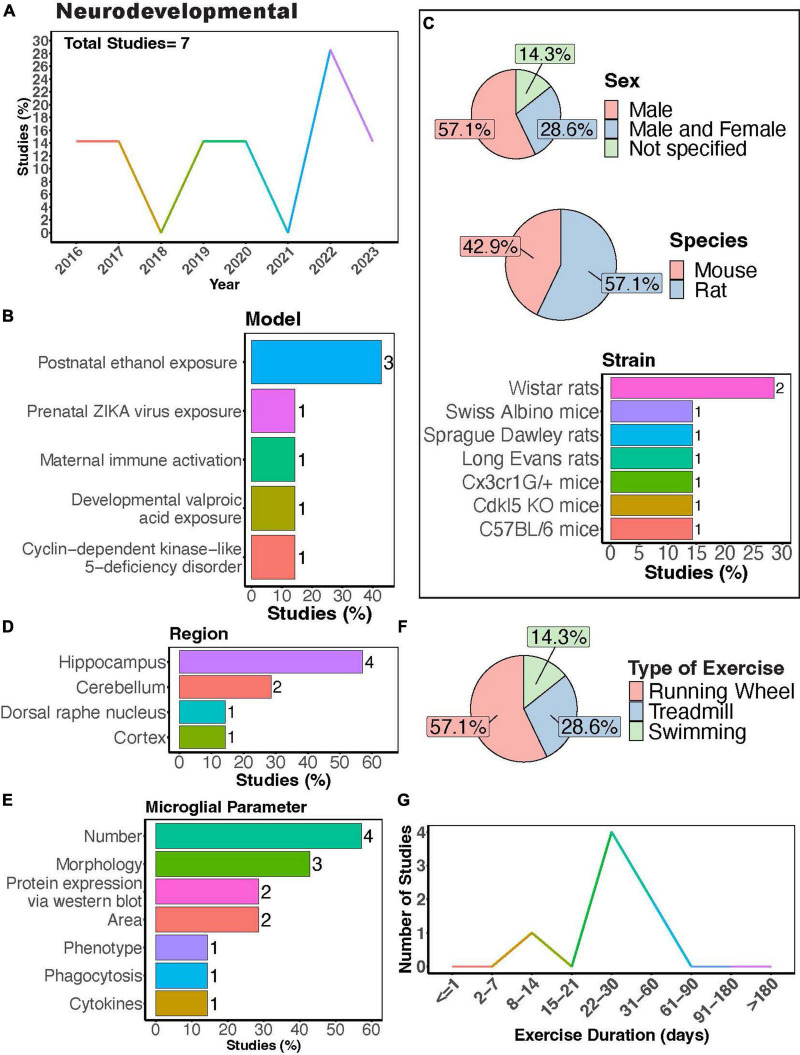
Physical exercise and microglia in neurodevelopmental models. **(A)** Percent studies published by year examining effects of physical exercise on microglia in neurodevelopmental models. Total number of studies = 7. **(B)** Percent of studies using various neurodevelopmental models. **(C)** Percent of studies utilizing male, female, or both sexes (top), various species (middle), and strains (bottom). **(D)** Percent of studies which examine microglia in the brain regions shown. **(E)** Percent of studies which measured microglia parameters shown. **(F)** Percent of studies utilizing different types of exercise paradigms. **(G)** Number of studies implementing exercise paradigms of various durations. Numbers of studies are included next to the bars for percentage plots. For **(C–E)**, percentages exceed 100% as some studies used multiple strains, measured more than one microglial parameter, or examined more than one brain region.

Microglial play crucial roles in neurodevelopment, engaging in synaptic pruning, regulating neuronal viability and migration, as well as axonal sprouting (Paolicelli and Ferretti, 2017). Dysregulation of their activity is thought to contribute to the pathology of neurodevelopmental disorders. Recent meta-analyses show that physical exercise can improve executive function in children with atypical neurodevelopment (Shi et al., 2024) and reduce social disorders as well as repetitive behaviors in children with autism spectrum disorder (Wang S. et al., 2023). A modest number of studies have sought to understand how PE may regulate microglial function during the progression of neurodevelopmental disorders using rodent models (Figures 4A,B). Most of these studies have focused on neurodevelopmental outcomes in males (Figure 4C, 57.1%), with Wistar rats being the most utilized strain (Figure 4C). Most studies measured microglial parameters in the hippocampus, followed by the cerebellum (Figures 4D, E). Exercise inventions were conducted using running wheels, treadmills, or swimming (Figure 4F). Interestingly, Shariat et al. (2024) showed that aquatic exercises are effective at improving motor and social skills in children with neurodevelopmental disorders, making the effects of swimming intervention on microglial activity of interest. One study investigated the effectiveness of a swimming intervention in a mouse model of prenatal Zika virus exposure, finding swimming exercise during ZIKA exposure during pregnancy prevented behavioral defects, brain atrophy, and microglial reactivity in the hippocampus (De Sousa et al., 2022). Most studies implemented exercise protocols for 1 month (Figure 4G), although one study utilized a 12-day exercise regimen (Gursky et al., 2020) and two implemented exercise protocols for approximately 2 months (Vetreno et al., 2017; Guo et al., 2022).

### Physical exercise and microglia in fetal alcohol spectrum disorders

There is evidence suggesting that exercise may offer benefits to children with fetal alcohol spectrum disorders (FASD), as improvements in executive function have been observed following exercise intervention, persisting for up to 3 months post-intervention (Pritchard Orr et al., 2018). Microglia have been suggested to play a role in FASD pathology although this is still under active investigation. In mice, microglia dynamics appear to be minimally affected in both the cortex (Wong et al., 2021) and lobule 4/5 of the cerebellum (Cealie et al., 2023) in a third-trimester equivalent mouse alcohol exposure model of FASD. However, Gursky et al. showed that a similar alcohol exposure increased microglial density and reduced ramification in lobules 1-4 of the cerebellum. Additionally, alcohol-exposed mice exercising for 12 days had decreased microglial density and increased number of ameboid microglia in lobules 1-4 of the cerebellum (Gursky et al., 2020), demonstrating the potential for exercise to reverse some microglial effects in models of alcohol exposure. Furthermore, exercising for 2 months can counteract adolescent intermittent alcohol exposure-induced increases in microglial number (Vetreno et al., 2017), morphological activation (Guo et al., 2022), and pro-inflammatory cytokine production (Guo et al., 2022). Further studies on how PE may regulate microglial function in FASD models could help uncover the therapeutic potential of exercise in FASD.

### Physical exercise and microglia in other models of neurodevelopmental disorders

Other rodent models of neurodevelopmental disorders have studied exercise effects on microglia, including developmental valproic acid exposure, cyclin-dependent kinase-like 5-deficiency disorder, and maternal immune activation (Figure 4B). In a developmental valproic acid exposure model, Cho et al. (2016) found that treadmill exercise after birth for 1 month ameliorated motor dysfunction and inhibited microglial reactivity in the cerebellum. In a mouse model of cyclin-dependent kinase-like 5-deficiency disorder—a developmental encephalopathy resulting from genetic mutations in the CDKL5 gene—VWR for 1 month in adulthood improved behavioral outcomes and neurogenesis, while also preventing increases in microglial density and cell body size (Mottolese et al., 2023). Furthermore, Andoh et al. (2019) found that VWR in adulthood could reverse behavioral and synaptic deficits in offspring after maternal immune activation, probably by enhancing microglial pruning in the hippocampus. These changes were observed in the absence of changes in the density of microglia or CD68 volume. This highlights the ability of exercise to stimulate microglial phagocytic activity, which could be beneficial in neurodevelopmental diseases where microglia fail to prune synapses. Together these studies provide evidence that exercise may be beneficial in counteracting changes in microglia function associated with neurodevelopmental deficiencies.

### Novel avenues to explore the role of physical exercise in neurodevelopmental models

There are several other mouse models of autism that could also been used to test the effects of physical activity on microglial function. It would be interesting to test whether exercise intervention in adulthood would also be useful in the Neuroligin-3 (NL3^*R*451*C*^) mouse model of autism, where microglial density, morphology and injury response has been shown to be altered (Matta et al., 2020; Guneykaya et al., 2023). It may also be interesting to test the effectiveness of exercise intervention in multiple ankyrin repeat domains 3 (Shank3) mutant mice, which model autism spectrum disorder, Phelan-McDermid Syndrome, and schizophrenia. Shank3 is an abundant excitatory post-synaptic scaffolding protein and mutant mice show synaptic and behavioral deficits. Microglia exhibit a sex specific expression of Shank 3, with lower expression in male microglia compared to female microglia (Villa et al., 2018). Microglia morphology and density are reported to be unaltered in adult Shank3 mutant mice (Cope et al., 2016). However, a newer report shows changes in transcriptomic expression of microglial genes in several regions between juvenile and adult Shank3 mutant mice (Yoo et al., 2022). It is therefore possible that microglia may have altered functions at different developmental stages in this model. Whether physical activity could counter some of these microglial changes in these models have not yet been tested.

It is also possible that the benefits of exercise could extend to other neurodevelopmental diseases, such as fragile X syndrome. Fragile X syndrome is the most common cause of inherited intellectual disability caused by hypermethylation of the Fmr1 gene, which impairs translation of Fragile X messenger ribonucleoprotein 1 protein (FMRP). Exercise can regulate FMRP expression in wild type mice (Yan et al., 2023) and stimulate hippocampal neurogenesis in FMRP-/- mice (Pinar et al., 2018). However, no studies have reported the effects of exercise on microglia activity in a mouse model of fragile X. Moreover, exercise may serve as a beneficial intervention for Rhett syndrome, a severe disorder that is caused by the loss of function of X-linked methyl-CpG-binding protein 2 (Mecp2). In humans, some reports suggest treadmill walking may benefit females with Rett syndrome (Larsson et al., 2018; Stahlhut et al., 2020). These results are mirrored in mouse models of Rhett syndrome, where forced exercise improved coordination and anxiety in Mecp2-null mice (Yue et al., 2021). Microglia have been implicated in the pathogenesis of Rett syndrome in MECP2-null mice both in the early (Zhao et al., 2017) and end stages of the disease (Schafer et al., 2016). Therefore, testing different durations and timing of exercise intervention in this model could help uncover whether benefits of exercise may be tied to changes in microglial activity. Altogether, knowledge on the effects of physical activity in neurodevelopmental model remain limited and there is great opportunity for further research.

## Physical exercise regulates microglia during aging

As the population continues to age, understanding how PE may regulate microglia during aging holds significant value. In elderly humans, increasing evidence indicates that higher levels of PE can regulate microglial morphology, potentially predicting changes in synaptic protein expression or cognitive function (Casaletto et al., 2022). There is a consensus that cognitive function declines without widespread neuronal loss during healthy aging (Gómez-Isla et al., 1996; Freeman et al., 2008), contrasting observations in neurodegenerative diseases where cognitive deficits are associated with synaptic loss and abnormalities. In congruence with this, microglial phenotypes observed in aging are thought to be distinct from those seen in neurodegenerative diseases. In the aging rodent brain, microglia become “primed” with an exaggerated inflammatory response (Perry and Holmes, 2014) and shift to more pro-inflammatory phenotype characterized by increases in MHCII, CD68, CD86, and complement receptor 3 (CR3) expression (Perry et al., 1993; Kohman et al., 2013; Giorgetti et al., 2019), rendering them more sensitive to insults or stimuli. An example of this “primed” response to insult was demonstrated in stroke models, where aged microglia exhibited distinct differences in expression of interferon regulatory factors 4 and 5 *in vivo* (Ngwa et al., 2022) and enhanced phagocytosis capacity and more cytoplasmic inclusions *in vitro* (Ngwa et al., 2021) following stroke. Furthermore, microglia in aged mice exhibit differences in morphology and number compared to adult mice, mirroring changes observed in post-mortem human samples (Davies et al., 2017; Edler et al., 2021). Generally, microglia display less ramified processes with increased cell soma size with age (Perry et al., 1993; Hefendehl et al., 2014; Davies et al., 2017; Tejera et al., 2019). Such decreases in microglial process ramification have been reported in multiple brain regions with age, including the cortex (Hefendehl et al., 2014; Davies et al., 2017; Tejera et al., 2019), hippocampus (Aires et al., 2021), and retina (Damani et al., 2011; Choi et al., 2022). Changes in microglia numbers and distribution with age are conflicting, with some studies reporting increases in microglia density in the hippocampus (Mouton et al., 2002), cortex (Tremblay et al., 2012) and retina (Damani et al., 2011), while others report no differences (Long et al., 1998; Choi et al., 2022), and some show decreases (Hayakawa et al., 2007; Adachi et al., 2010). In the spinal cord, the overall microglial cell area was increased in aged mice with a non-significant trend toward increased cell numbers (Giorgetti et al., 2019). The substantia nigra follows a similar trend, with age-related increases in microglial area and number (Wang T. F. et al., 2022). Microglial dynamics also change with age and by region. In the cortex, microglia process motility decreases with age, while soma movement increases (Hefendehl et al., 2014). Similar age-related decreases in microglial motility have been observed in the retina (Damani et al., 2011). In the hippocampus, microglia process surveillance decreases in aged mice, with no alterations in motility (Aires et al., 2021). Overall, there is strong evidence that aged microglia function differently than younger microglia, and regulating or preventing some of these morphological, dynamic, and phenotypic changes could be used in the treatment of age-related neurocognitive diseases.

**FIGURE 5:**
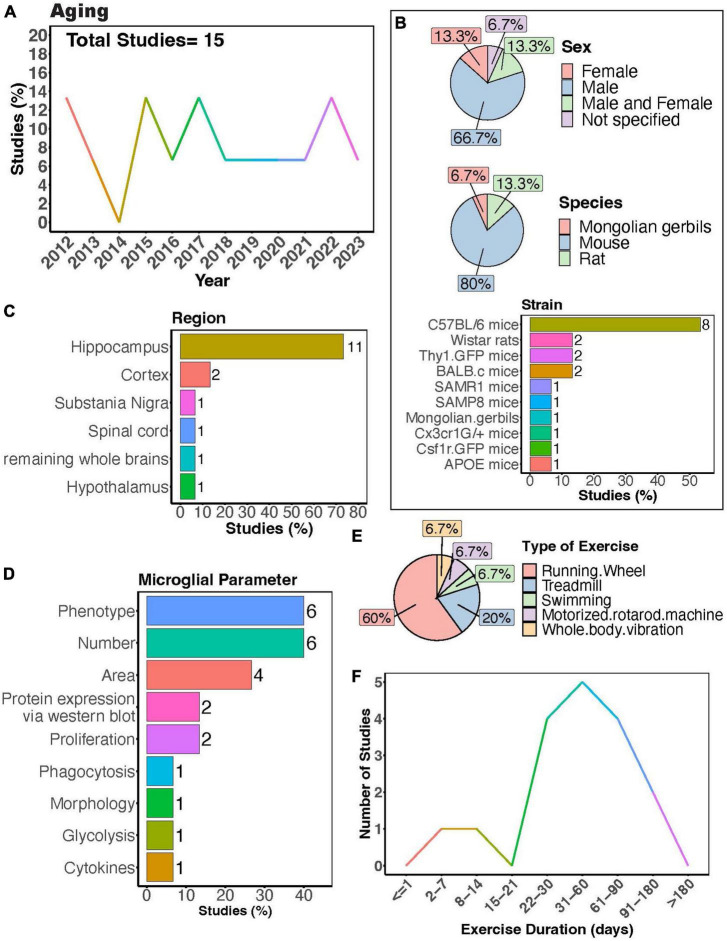
Physical exercise regulates microglia during aging. **(A)** Percent studies published by year examining effects of physical exercise on microglia during aging. Total number of studies = 15. **(B)** Percent of studies utilizing male, female, or both sexes (top), various species (middle), and strains (bottom). **(C)** Percent of studies which examine microglia in the brain regions shown. **(D)** Percent of studies which measured microglia parameters shown. **(E)** Percent of studies utilizing different types of exercise paradigms. **(F)** Number of studies implementing exercise paradigms of various durations. Numbers of studies are included next to the bars for percentage plots. For **(B–D)**, percentages exceed 100% as some studies used multiple strains, measured more than one microglial parameter, or examined more than one brain region.

The interest in understanding how PE can regulate microglial activity during aging has remained relatively steady (Figure 5A). Many studies exclusively used males (Figure 5B, > 65%), again underscoring the necessity for more research including females. Among these studies, the majority employed mouse models, with C57BL/6 mice being the most utilized strain (Figure 5B). The overwhelming majority of studies investigated microglial parameters in the hippocampus (Figure 5C, > 70%), highlighting a significant gap in our understanding of how exercise may modulate aged microglial activity in other brain regions (Figures 5C,D). Most studies utilized a running wheel as the form of exercise, where most subjects underwent exercise for a duration of 1–2 months (Figures 5E,F). PE can regulate the phenotype of microglia in aged mice across various brain regions, including the spinal cord, hippocampus, and cortex (Kohman et al., 2012, 2013; Littlefield et al., 2015; Soto et al., 2015; Giorgetti et al., 2019; Mela et al., 2020). These changes encompass decreased expression of activation markers such as CD86 and MHC II (Kohman et al., 2013) as well as components of the complement pathway (Soto et al., 2015), alongside increased expression of neurotrophic factors like IGF-1 and BDNF in aged microglia (Kohman et al., 2012; Littlefield et al., 2015). Furthermore, exercise can modulate age-related changes in inflammation (interleukin 1- beta (IL-1β)) and signs of senescence (as evidenced by expression of β-Galactosidase and p16^*INK*4*A*^) (Mela et al., 2020). Additionally, Mela et al. (2020) found that microglia isolated from aged mice that exercised exhibited altered phagocytic capacity and reduced glycolysis. In aged mice, longer durations of physical exercise ranging from 5 weeks to 6 months consistently reduce numbers of microglia in the hippocampus (Kohman et al., 2012, 2013; Littlefield et al., 2015; He et al., 2017), cortex (Soto et al., 2015; He et al., 2017), and substantia nigra (Wang T. F. et al., 2022). However, two studies implementing an exercise duration of 4 weeks reported no changes in microglial numbers in the hippocampus of aged mice (Vukovic et al., 2012; Singhal et al., 2021). Collectively, these findings indicate a strong effect of exercise on microglial function during aging. Whether exercise can mitigate age-induced changes in microglial soma and process dynamics remains to be determined.

Moreover, exercise has been demonstrated to regulate microglial responses to secondary insults in aged rodents. For instance, exercise prevented *E. coli* infection-induced inflammatory cytokine production and age-related priming in rats (Barrientos et al., 2011). Littlefield et al. found exercise increased the proportion of BDNF+/ ionized calcium-binding adapter molecule 1 (Iba1)+ cells in the hippocampus of aged mice, even in mice subjected to LPS administration (Littlefield et al., 2015). This suggests strong beneficial effects of exercise even in the presence of secondary injury. Microglial morphology has also been shown to be influenced by interactions between age, enriched environments containing running wheels, and Piry viral infection (de Sousa et al., 2015). However, some reports show aged microglia have a dampened injury response to laser ablation in the cortex (Hefendehl et al., 2014; Brawek et al., 2021) and retina (Damani et al., 2011), implying that a decreased response to injury might contribute to exacerbated pathology as individuals age. Whether exercise can ameliorate these differential responses of microglia to different insults with age should be examined.

## Physical exercise regulates microglia in neurodegenerative models

**FIGURE 6:**
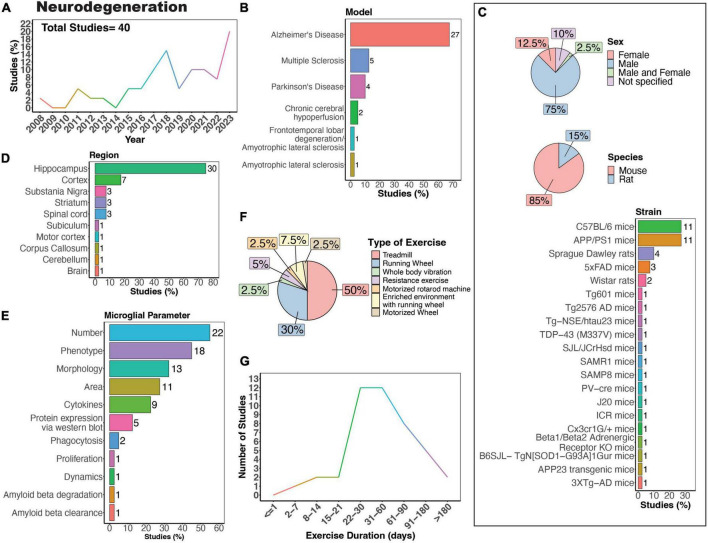
Physical exercise and microglia in neurodegenerative diseases. **(A)** Percent studies published by year examining effects of physical exercise on microglia in neurodegenerative models. Total number of studies = 40. **(B)** Percent of studies using models of various neurodegenerative diseases. **(C)** Percent of studies utilizing male, female, or both sexes (top), various species (middle), and strains (bottom). **(D)** Percent of studies which examine microglia in the brain regions shown. **(E)** Percent of studies which measured microglia parameters shown. **(F)** Percent of studies utilizing different types of exercise paradigms. **(G)** Number of studies implementing exercise paradigms of various durations. Numbers of studies are included next to the bars for percentage plots. For **(C–E)** percentages exceed 100% as some studies used multiple strains, measured more than one microglial parameter, or examined more than one brain region.

Microglia are pivotal in both the development and progression of neurodegenerative disease. When in a reactive state, microglia aid in clearing debris, phagocytose and eliminate protein aggregates, and offer neurotrophic support. Dysfunction in these processes can lead to the accumulation of toxic protein aggregates, further exacerbating neurodegeneration. However, prolonged activation of microglia can trigger chronic neuroinflammation, marked by the release of pro-inflammatory cytokines and reactive oxygen species, thereby promoting neurodegeneration. Notably, “reactive,” ameboid microglia have been observed in tissue from human patients with many neurodegenerative diseases (McGeer et al., 1988), including Huntington’s disease (HD) (Vonsattel et al., 1985; Sapp et al., 2001), amyotrophic lateral sclerosis (ALS) (Brettschneider et al., 2012; Dols-Icardo et al., 2020), Alzheimer’s disease (AD) (Itagaki et al., 1989; Davies et al., 2017; Paasila et al., 2019; Franco-Bocanegra et al., 2021), Multiple sclerosis (MS) (Prineas et al., 2001; van Horssen et al., 2012; Singh et al., 2013; Kuhlmann et al., 2017), and Parkinson’s disease (PD) (Knott et al., 1999; Imamura et al., 2003). There has been a growing interest in understanding how PE may influence the activity of microglia in neurodegenerative diseases (Figure 6A). The effects of PE on microglia activity are well-studied in the context of neurodegeneration, particularly in rodent models of MS, AD and PD (Figure 6B). The majority of studies primarily used male mice (Figure 6C, 75%), which poses a problem considering that several neurodegenerative diseases, such as AD and MS, exhibit higher prevalence in females compared to males (Cao et al., 2020; Walton et al., 2020). Possible explanations for the higher experimental usage of males compared to females include historical sex-bias toward male animals in research, variability in experimental results due to the estrous cycle in females, and limited availability of transgenic models of both sexes. Most of these studies used mouse models (Figure 6C, 85%) employing various strains to model different neurodegenerative diseases and processes (Figure 6C). Although various brain regions have been investigated, most studies assessed microglial parameters in the hippocampus (Figures 6D, E, 75%). Treadmill exercise was predominantly employed (Figure 6F, 50%), closely followed by running wheels (Figure 6F, 30%). Additionally, most studies implemented exercise protocols lasting between 3 weeks and 6 months (Figure 6G).

### Physical exercise and microglia in Alzheimer’s disease

AD is the most common form of dementia, estimated to account for 60–70% of cases, and is characterized by tau and amyloid pathology leading to substantial cognitive impairment (World Health Organization, 2023). However, it is important to note that there are individuals with neuropathological features of AD that do not develop cognitive deficits (Bjorklund et al., 2012). In the case of AD, there is substantial evidence showing PE can exert beneficial effects on cognitive function (López-Ortiz et al., 2023). A recent systematic review of 21 studies showed exercise was associated with a lower risk of AD in humans (López-Ortiz et al., 2023). Physical exercise can also increase network connectivity in humans with mild cognitive impairment (Won et al., 2023). While numerous animal models of AD exist, no model recapitulates all aspects of AD pathology seen in humans. AD animal models commonly utilize mutations in genes related to amyloid beta [amyloid protein precursor (APP), presenilin-1 (PSEN1), presenilin-2 (PSEN2)] and tau [microtubule-associated protein tau (MAPT)] processing, which lead to the formation of plaques and neurofibrillary tangles (Sanchez-Varo et al., 2022; Yokoyama et al., 2022). The predominant strain utilized to study the impact of physical exercise on microglia in AD in animals is the APP/PS1 mouse model (Figure 6C), in which transgenic mice express human mutant APP and PS1 (Jankowsky et al., 2004). In rodent models, many studies mirror the regulatory effect of exercise on AD pathology seen in humans. Nonetheless, there are some reports that physical exercise is ineffective in regulating AD pathology in 5XFAD, APP/PS1, and Tg2576 mouse models, all characterized by the presence of plaques in the absence of tau pathology (Nichol et al., 2008; Xu et al., 2013; Zhang J. et al., 2018; Belaya et al., 2020; Svensson et al., 2020). One study using female Tg601 mice, which overexpress the wild-type human tau sequence (2N4R), showed exercise promoted neuroinflammation by increasing the number Iba1-positive microglial cells and levels of inflammatory cytokines IL-1β and IL-18 in the hippocampus (Elahi et al., 2016). In terms of specific microglial parameters, conflicting evidence exists regarding the impact of exercise on microglial numbers in AD mice. Some studies suggest that exercise leads to a decrease in microglial numbers (Ke et al., 2011; Leem et al., 2011; Rodríguez et al., 2015; Wang Y. et al., 2023; Yang et al., 2023), while others indicate an increase (Elahi et al., 2016; Xu et al., 2016; Hashiguchi et al., 2020; Zhang S. et al., 2022; Campos et al., 2023), and some findings show no significant change in microglial density in AD mice (Xu et al., 2018; Zhang J. et al., 2018; Ziegler-Waldkirch et al., 2018; Oroszi et al., 2023). In terms of microglial phenotype, exercise can regulate CD68+ (Ziegler-Waldkirch et al., 2018; Zhang S. et al., 2022; Oroszi et al., 2023; Wang Y. et al., 2023), CD86+ (Lu et al., 2017; Zhang et al., 2019; Feng et al., 2023; Yang et al., 2023), triggering receptor expressed on myeloid cells 2 (TREM2) (Zhang L. et al., 2022) and inflammatory molecules (Xu et al., 2016, 2018; Nakanishi et al., 2021; Han et al., 2023) in AD rodent models. Microglia morphology can also be regulated by physical exercise in AD mice, with most studies reporting that exercise reduces the numbers of reactive microglia and increases process ramification (Ke et al., 2011; Leem et al., 2011; Xiong et al., 2015; Xu et al., 2016; Lu et al., 2017; Zhang S. et al., 2022; Feng et al., 2023; Oroszi et al., 2023). However, one study utilizing 3XTg-AD mice, which exhibit plaque pathology and tau pathology at later stages, showed that 9 months of exercise increased hippocampal microglial hypertrophy (microglia surface, volume and somata volume) (Rodríguez et al., 2015), indicating prolonged exercise may have differential regulatory effects compared to exercise of shorter durations. Interestingly, no studies have examined whether physical exercise can regulate the dynamic behavior of microglia in AD models. Such studies could help further uncover mechanisms through which exercise can regulate the function of microglia in the context of neurodegeneration.

### Physical exercise and microglia in multiple sclerosis

Exercising 60 min per day, 3 times or more per week, for 8–10 weeks can improve memory and cognitive function in MS patients (Li G. et al., 2023). Several rodent MS models are used to recapitulate different aspects of MS pathology. The cuprizone demyelination model is representative of the relapsing remitting form of MS present in most MS patients, whereas experimental autoimmune encephalomyelitis (EAE) is representative of chronic progressive MS (Ransohoff, 2012; Vega-Riquer et al., 2019). Demyelination, inflammation, microglial activation, astrogliosis, and behavioral disabilities are present in both cuprizone-treated and EAE mice (Ransohoff, 2012; Vega-Riquer et al., 2019). The cuprizone model, however, has a remyelination phase, whereas the EAE model consistently shows immune cell infiltration in the CNS (Ransohoff, 2012; Vega-Riquer et al., 2019). Though limited in number, studies show exercise can regulate microglia function in both cuprizone and EAE models using female mice (Mandolesi et al., 2019; Rizzo et al., 2021; Zaychik et al., 2021). However, in male mice exercise was unable to prevent cuprizone-induced increases in hippocampal microglia number (Naghibzadeh et al., 2018), indicating potential sex-differences in the effectiveness of exercise intervention. Mifflin et al., also found no effect of exercise on microglia in males or females using an EAE model (Mifflin et al., 2017). Further experimentation using both males and females as well as multiple types and durations of exercise could help uncover the effectiveness of exercise in MS models.

### Physical exercise and microglia in Parkinson’s disease

PD is the second most common neurodegenerative disease in the elderly population and is characterized by the loss of dopaminergic neurons and formation of Lewy bodies (Poewe et al., 2017). The number of people with PD over age 50 is expected to double between 2006 and 2030, creating an increasing need for effective therapeutic inventions (Dorsey et al., 2007). A recent meta-analysis shows that exercising at least 60 min per day is an effective intervention for enhancing global cognitive function and executive function in PD patients (Kim et al., 2023). However, the mechanisms behind these positive effects remain to be determined. PD is commonly modeled in rodents using exposure to toxicants, such as 1-methyl-4-phenyl-1,2,3,6-tetrahydropyridine (MPTP) or Rotenone. The MPTP and rotenone exposure models both replicate many features of PD, including microglial reactivity, nigrostriatal dopaminergic degeneration, and behavioral deficits (Betarbet et al., 2000, 2006; Du et al., 2001; Sedelis et al., 2001; Zhang J. et al., 2022). However, only the Rotenone exposure model exhibits α-synuclein accumulation and aggregation with formation of Lewy body-like inclusions, mimicking human PD, and this model also shows greater microglia area and numbers in the substantia nigra than the MPTP model (Betarbet et al., 2000, 2006; Zhang J. et al., 2022). Microglia numbers are generally shown to increase in the substantia nigra of PD models, with increases in morphological and phenotypic signs of reactivity (Sung et al., 2012; Gil-Martínez et al., 2018; Lee et al., 2018; Wang W. et al., 2021). While VWR has been reported to be ineffective (Gil-Martínez et al., 2018) in PD models, treadmill exercise is successful in preventing the increase in microglial cell numbers seen in these models (Sung et al., 2012; Lee et al., 2018; Wang W. et al., 2021). One possible explanation is that treadmill running offers a more controlled environment, where researchers can precisely adjust factors such as speed, duration, and incline, which may all impact outcomes. Notably, there is a lack of information on how exercise may impact PD pathology in females, as these studies all used male mice. As women develop PD, and in fact, may experience greater disease severity (Dahodwala et al., 2016), research is needed to discern the effectiveness of exercise in female PD models.

### Physical exercise and microglia in Huntington’s disease

Nevertheless, PE may not be universally beneficial for all neurodegenerative diseases. HD is a genetically inherited disease caused by a mutation in the gene encoding the Huntington protein which leads to progressive cognitive decline manifesting in involuntary motor movements and its progression does not appear to be sensitive to physical exercise. A systematic review of seventeen studies in humans examining the effects of exercise and cognitive interventions found that exercise intervention may be negligible in HD, even when combined with cognitive interventions (Huynh et al., 2023). Another report showed an absence of cortical plasticity in response to an acute bout of cardiorespiratory exercise in premanifest and early HD patients (Andrews et al., 2022). Though some rodent models of HD have shown that VWR (Pang et al., 2006; van Dellen et al., 2008; Harrison et al., 2013) or treadmill exercise (Stefanko et al., 2017; Caldwell et al., 2020) can improve cognitive outcomes, in one study VWR surprisingly accelerated disease onset in male N171-82Q transgenic HD mice (Potter et al., 2010). Though microglia are believed to facilitate early neuroinflammatory processes in HD patients (Palpagama et al., 2019), clear evidence showing PE can regulate microglial function to alter the manifestation and progression of HD remains to be established, as none of these animal studies examined microglia in the context of physical activity intervention.

### Physical exercise and microglia in amyotrophic lateral sclerosis

Strikingly, exercise may be harmful for some neurodegenerative diseases, as is the case for ALS. ALS is the most common motor neuron disease, and it is both rapidly progressive and fatal. A systematic review of ninety-three studies found strenuous anaerobic exercise (such as soccer, long-distance skiing and American football) was a risk factor for ALS (Chapman et al., 2023). The underlying mechanisms behind this remain elusive, though oxidative stress and dysregulated energy metabolism were highlighted as possible mediators of motor neuron stress and degeneration in ALS (Chapman et al., 2023). Microglia are linked to the development of motor neuron pathology in ALS patients (Cooper-Knock et al., 2017). In a mouse model of ALS, exercise increased microglial reactivity, shown by changes in morphology (hypertrophic, intensely stained microglia with thick and stout processes) (Kassa et al., 2017), further supporting the notion that exercise may not be beneficial in treating ALS. Nevertheless, there is some evidence suggesting that exercise can beneficially modulate microglia dynamics. Mutations in the TAR DNA binding protein 43 kDa (TDP-43) are observed in frontotemporal lobar degeneration and ALS and are thought to be partially mediated by microglia dysfunction. A recent study by Wei et al. revealed that microglia in TDP-43 mutant mice exhibited enhanced phagocytic activity and dysregulated soma and process dynamics (Wei et al., 2023). Specifically, TDP-43 induced higher soma migration distances, reduced microglial process territory, increased process velocity, and increased the fraction of retracted processes over an hour-long imaging session (Wei et al., 2023). Two weeks of treadmill exercise at the pre-symptomatic stage restored normal microglial dynamics, reduced CD68 expression, restored morphology changes, and improved motor learning of mutant TDP-43 mice (Wei et al., 2023). This demonstrates the capacity of exercise to regulate microglial dynamics and prevent cognitive dysfunction in a model of ALS, though further research is needed to explore these effects. Determining how PE differentially regulates microglia in contexts where PE may be ineffective or harmful (HD or potentially ALS), compared to those where PE is reported to be helpful (AD, MS, PD) could provide useful insights into new therapies for neurodegenerative disorders.

## Physical exercise regulates microglia function in stroke models

**FIGURE 7:**
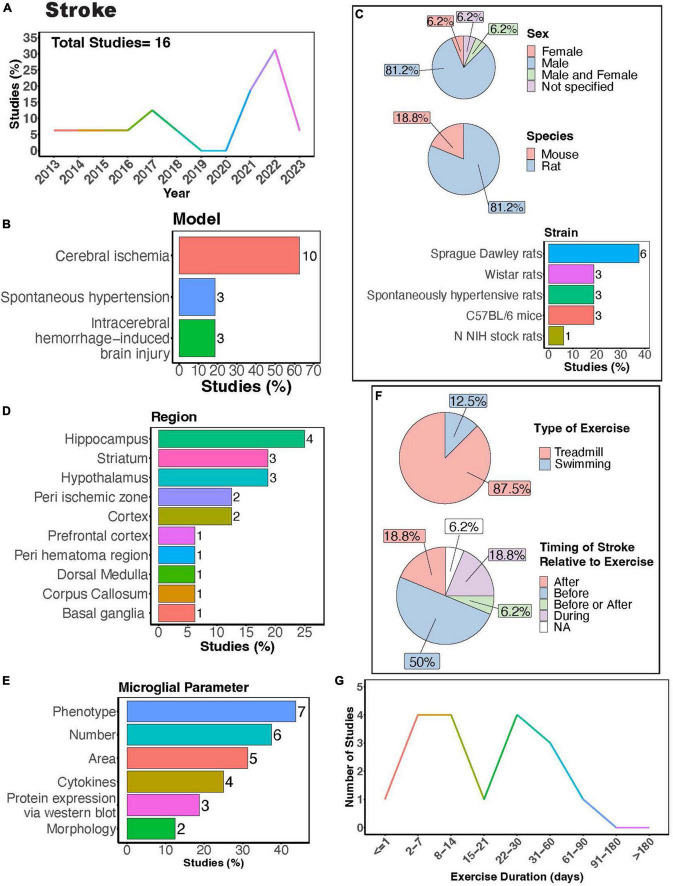
Physical exercise regulates microglia in stroke models. **(A)** Percent studies published by year examining effects of physical exercise on microglia in stroke models. Total number of studies = 16. **(B)** Percent of studies using different stroke models. **(C)** Percent of studies utilizing male, female, or both sexes (top), various species (middle), and strain (bottom). **(D)** Percent of studies which examine microglia in the brain regions shown. **(E)** Percent of studies which measured microglia parameters shown. **(F)** Percent of studies utilizing treadmill or swimming exercise (top) and the timing of stroke relative to exercise intervention (before, after, before or after, during, not specified; bottom). Studies where exercise intervention occurs “during” stroke utilized spontaneous hypertensive rats, which exhibit genetically induced increased blood pressure and as such the “stroke” occurred “during” the exercise intervention **(F)**. **(G)** Number of studies implementing exercise paradigms of various durations. Numbers of studies are included next to the bars for percentage plots. For **(D,E)** percentages exceed 100% as some studies used measured more than one microglial parameter or examined more than one brain region.

In humans, exercise can improve cognitive function and motor coordination among patients with cognitive impairments after stroke (Li W. et al., 2023). In stroke models, microglia play a complex role- they can promote neuroinflammation, thereby perpetuating damage, yet they can also release anti-inflammatory factors that facilitate repair (Wang Y. et al., 2022). Studies investigating how exercise can regulate microglia functions in stroke often employ animal models of cerebral ischemia, spontaneous hypertension, and intracerebral hemorrhage (ICH) (Figures 7A,B). Notably, a significant proportion of these studies focused solely on males (Figure 7C, > 80%), indicating a clear research gap concerning females. Rats were predominantly used in these studies (Figure 7C, > 80%), with Sprague Dawley rats being the most frequently utilized (Figure 7C). Microglial parameters were most frequently examined in the hippocampus, followed by the striatum and hypothalamus (Figures 7D,E).

Many of these investigations explored how exercise modulates microglial number and phenotype in stroke models (Figure 7E). Treadmill exercise has been shown to influence microglial phenotype after ischemia across various brain regions, including the striatum, corpus callosum, cortex, basal ganglia, and peri-ischemic and peri-hematoma zones (Kinoshita et al., 2021; Lu et al., 2021; Liu et al., 2022; Tamakoshi et al., 2022; Xu et al., 2023). Furthermore, a transgenerational study selecting for low capacity and high-capacity runners found male low-capacity runners had more severe ICH-induced brain injury and greater numbers of major histocompatibility complex class 2 1a (OX-6) positive microglia cells, demonstrating transgenerational regulation of microglia phenotype in a stroke model (He et al., 2013). Both treadmill and swimming exercise have been found to regulate microglial numbers in different brain regions, such as the hypothalamus, hippocampus, basal ganglia, as well as in peri-infarcted and peri-hematoma zones (Kinoshita et al., 2021; Xia et al., 2021; Aguilar-Peralta et al., 2022; Li et al., 2022). However, a single study by Svensson et al. reported no impact of exercise on hippocampal microglial number or phenotype in a cerebral ischemic stroke model (Svensson et al., 2016). Moreover, studies consistently found a decrease in microglia area with exercise intervention in stroke models (Lovatel et al., 2014; Buttler et al., 2017; Zhang et al., 2017; Zhang M. et al., 2018; Gao et al., 2022). Investigations into cytokine levels in stroke models with exercise intervention consistently report reductions in pro-inflammatory cytokines, such as tumor necrosis factor alpha (TNF-α), IL-1β, and interleukin 6 (IL-6) (Masson et al., 2015; Lu et al., 2021; Xia et al., 2021; Gao et al., 2022). Exercise also shifts microglial morphology towards a homeostatic state, characterized by increased process ramification (Li et al., 2022) and length, as well as reduced microglial cell size (Xia et al., 2021). While there are reports indicating altered microglial process motility in ischemic stroke (Morrison and Filosa, 2013), no studies have yet explored how exercise intervention might influence these dynamic properties of microglia.

Treadmill exercise was the predominantly used form of PE, with fewer studies utilizing swimming (Figure 7F). This highlights a knowledge gap regarding the potential of voluntary interventions, such as those employing a running wheel, to modulate microglia function. Given that VWR is perceived as less stressful and mirrors a more natural rodent behavior, delving into this paradigm is crucial. Most studies implement injury or ischemia prior to exercise intervention (Figure 7F), thus limiting our understanding of potential differences in responses between individuals who were previously active versus sedentary before injury. Exercise durations in these studies vary widely, ranging from 1 to approximately 90 days (Figure 7G). Notably, only one study implemented an exercise intervention lasting longer than 2 months. Consequently, future research is necessary to determine whether individuals who engage in regular exercise throughout their lifespan exhibit distinct responses to stroke compared to those leading predominantly sedentary lifestyles.

## Exercise interacts with lifestyle factors to modulate microglial activity

It is believed that lifestyles factors, such as exercise, diet, stress, alcohol consumption, and toxicant exposure, influence brain health and cognitive function. Adopting a healthy lifestyle can play a crucial role in reducing neuroinflammation and lowering the risk of developing neurodegenerative and psychiatric disorders (Kip and Parr-Brownlie, 2023). A recent report by Dhana et al. (2024) showed that adopting a healthy lifestyle may facilitate the maintenance of cognitive abilities in older adult). In this context, a healthy lifestyle was characterized by physically activity, eating healthy (increasing green leafy vegetables, nuts, berries, beans, whole grains, seafood, poultry relative to red meats, butter, cheese, sweets, and fried food), and avoiding smoking and limiting alcohol intake (Dhana et al., 2024). How these lifestyle factors interact to influence microglial activity, which can in turn regulate neuronal health, is largely unknown.

**FIGURE 8:**
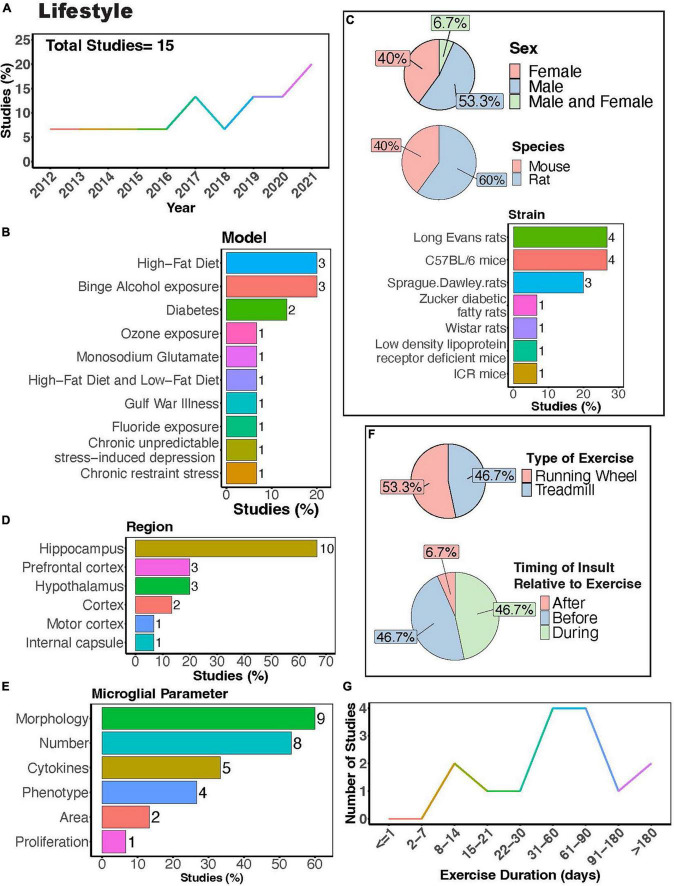
Physical exercise interacts with lifestyle factors to modulate microglial activity. **(A)** Percent studies published by year examining effects of physical exercise and lifestyle factors on microglia activity. Total number of studies = 15. **(B)** Percent of studies using different lifestyle models. **(C)** Percent of studies utilizing male, female, or both sexes (top), various species (middle), and strains (bottom). **(D)** Percent of studies which examine microglia in the brain regions shown. **(E)** Percent of studies which measured microglia parameters shown. **(F)** Percent of studies utilizing treadmill or running wheel exercise (top) and the timing of insult (stress, alcohol, environmental, dietary exposure) relative to exercise intervention (before, after, during; bottom). **(G)** Number of studies implementing exercise paradigms of various durations. Numbers of studies are included next to the bars for percentage plots. For **(D,E)** percentages exceed 100% as some studies used measured more than one microglial parameter or examined more than one brain region.

In recent years, interest in the influence of physical exercise on microglial activity in the context of diet, alcohol, stress, and toxicant exposure has grown (Figure 8A). Numerous studies have investigated how physical exercise regulates microglial activity in rodent models of toxicant exposure (Valdez et al., 2020; Kodali et al., 2021; Wang J. et al., 2021), binge alcohol exposure (Barton et al., 2017a,b; West et al., 2019), diet (Yi et al., 2012; Lima et al., 2014; Yoo et al., 2015; Kang et al., 2016; Yin et al., 2018; Klein et al., 2019; Lang et al., 2020), and chronic stress (Gerecke et al., 2013; Xiao et al., 2021; Figure 8B). Most of these studies primarily used male subjects, though females were examined more often than in studies that focused on disease outcomes (Figure 8C). Many investigations utilized rat models (Figure 8C, 60%), with Long-Evans rats and C57BL/6 mice being the most common strains (Figure 8C). Most studies focused on evaluating microglial parameters in the hippocampus (Figures 8D, E, 67%) and cortical areas. Exercise interventions were conducted using running wheels (Figure 8F, 53.3%) or treadmills (Figure 8F, 46.7%), and most studies implemented exercise protocols lasting between 1 and 3 months (Figure 8G). Additionally, it is important to consider the timing of exercise intervention relative to the dietary, alcohol, stress, or toxicant exposure. Most studies implemented exercise protocols before or during the diet, alcohol, stress, or toxicant exposure (Figure 8F). This reveals a scarcity of literature exploring the potential benefits of exercise intervention after exposure to stress, alcohol, dietary, or toxicants.

Rodent models investigating effects of diet and exercise on microglia activity include high-fat diet or low-fat diet (Yi et al., 2012; Kang et al., 2016; Yin et al., 2018; Klein et al., 2019), diabetes models (Yoo et al., 2015; Lang et al., 2020) as well as treatment with monosodium glutamate (Lima et al., 2014) (MSG; Figure 8B). Exercise can attenuate high-fat diet-induced microglial reactivity in the hypothalamus, white matter internal capsule, hippocampus, and cortex (Yi et al., 2012; Kang et al., 2016; Yin et al., 2018; Klein et al., 2019). Although no significant interaction between MSG and exercise was observed in rats, both MSG treatment and exercise increased microglial Iba1+ area in the motor cortex (Lima et al., 2014). In diabetes models, exercise reduced microglial morphological changes, their number, and pro-inflammatory cytokine production (Yoo et al., 2015; Lang et al., 2020). In models of binge alcohol exposure, there is conflicting evidence on the ability of exercise to regulate microglial numbers and morphology, with some reports showing that exercise can regulate these parameters (Barton et al., 2017b; West et al., 2019) while a study by Barton et al. showed no effects on these parameters (Barton et al., 2017a). Nonetheless, Barton et al. did show a significant interaction between binge alcohol exposure and physical exercise, with exercise increasing the number of MHC II+ microglia in female mice exposed to binge alcohol, demonstrating the ability of exercise to regulate microglial phenotype in this model (Barton et al., 2017a). Overall, these studies suggest exercise can interact with dietary exposure to influence microglial activity.

Stress is thought to play a pivotal role in both the development and maintenance of neuropsychiatric disorders like major depression, anxiety disorders, and post-traumatic stress disorder, and is often used to model these conditions in rodents. Although limited, there is evidence that physical exercise can regulate microglia activity in models of chronic stress. Exercise can protect against stress-induced increases in microglial expression of CD68 and Cyclooxygenase 2 (Cox-2), demonstrating the capacity of exercise to induce phenotypic changes in microglial response to stress (Gerecke et al., 2013; Xiao et al., 2021). Xiao et al. also found that exercise attenuated the stressed-induced increases in the number of microglia and pro-inflammatory cytokine IL-1β production in the hippocampus (Xiao et al., 2021). Together these studies suggest that exercise can alleviate stress-induced alterations in microglial function. However, stress can be induced using diverse methods, including models of social stress or non-social stress (such as chronic-restraint stress employed by Gerecke et al., 2013), administered for various durations. It remains to be determined how PE may regulate microglia in these various models of stress and whether these changes are sustained over time.

While environmental toxicants can modulate microglia activity, inducing changes in dynamics (Lowery et al., 2022) and morphology (Yi et al., 2012) indicative of classical microglial reactivity, very few studies have examined exercise regulation of microglia activity in response to toxicant exposure. The models that have include fluoride (Wang J. et al., 2021) and ozone (Valdez et al., 2020) exposure, as well as a model of Gulf war illness, which encompassed daily exposure to mosquito-repellant N, N-diethyl-m-toluamide (DEET), insecticide permethrin (PER), and nerve gas prophylactic drug pyridostigmine bromide (PB), accompanied by 15 min of restraint stress for 4 weeks (Kodali et al., 2021; Figure 8B). Wang J. et al. (2021) found that repeated treadmill running attenuated the morphological changes of microglia in the hippocampus of fluoride-exposed mice. Kodali et al. (2021) found that VWR reverses hippocampal microglia morphological changes in a mouse model of Gulf War illness. Both studies highlight the capability of exercise to prevent adverse effects caused by toxicant exposure. In contrast, Valdez et al. found that exercise did not prevent ozone (O3)-induced (1 ppm O3) microglial morphological changes in the hippocampus and the hypothalamus (Valdez et al., 2020). Notably, these studies were restricted to the hippocampus and hypothalamus, suggesting the necessity for exploration of other brain regions in future investigations. Future studies should also examine effects from a broader range of toxicants, considering that individuals are exposed to numerous toxic substances over the course of their lifetimes. For instance, lead (Pb) exposure is known to trigger microglial reactivity and neurological impairment (Liu et al., 2012; Wu et al., 2021; Shvachiy et al., 2023). One proposed mechanism of Pb toxicity involves the activation of nucleotide-binding oligomerization domain, leucine rich repeat and pyrin domain containing (NLRP) and the inflammasome system, an important component of the innate immune response triggered by exposure to other toxicants (Moloudizargari et al., 2019; Su et al., 2021). PE has been shown to regulate the inflammasome system and NLRP expression (Tang et al., 2022). Thus, it is possible that exercise could either prevent or attenuate some of the adverse effects on microglia activity resulting from Pb exposure or other toxicants acting through this pathway. Overall, very little is known regarding PE regulation of microglia activity relative to the vast number of toxicants known to impact brain health.

## Exercise increases known modulators of microglia activity

**FIGURE 9:**
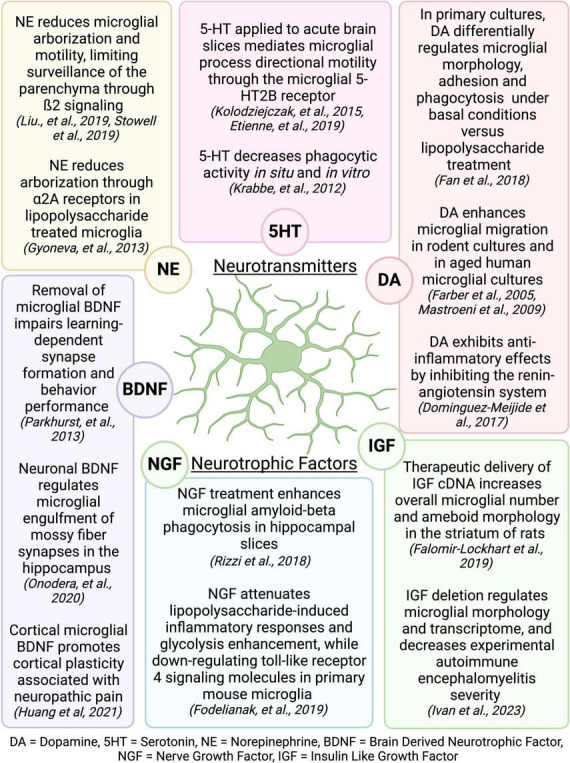
Neurotransmitters and neurotrophic factors that are increased during exercise are known regulators of microglial activity. Figure made with Biorender.com.

The mechanisms by which exercise elicits microglial changes are unknown, but it is likely that microglia respond to exercise-induced alterations in signaling molecules that broadly affect the function of many brain cell types. The benefits of PE have largely been attributed to increased production of neurotropic factors and enhanced neurogenesis in the hippocampus (van Praag et al., 1999a,b; Olah et al., 2009; Szuhany et al., 2015), which are extensively reviewed elsewhere (Meeusen and De Meirleir, 1995; Vecchio et al., 2018). Interestingly, many of the neurotransmitters and neurotrophic factors that are increased or regulated during PE are known modulators of microglial activity (Figure 9; Vecchio et al., 2018; Albertini et al., 2020). Exercise increases neurotrophic factors in both mice and humans, including those that influence cognition such as BDNF, IGF, and nerve growth factor (NGF) (Ding et al., 2006; Arvidsson et al., 2018; Żebrowska et al., 2018; Kang et al., 2020). Increases in BDNF with exercise are detected in the hippocampus and cortex (Russo-Neustadt et al., 1999; Adlard et al., 2004; Ploughman et al., 2005; Chen and Russo-Neustadt, 2009), where BDNF can regulate microglia-neuronal interactions, thereby influencing synapse formation and removal (Parkhurst et al., 2013; Huang et al., 2021; Onodera et al., 2021). IGF also increases with exercise in the hippocampus, motor cortex, and striatum (Carro et al., 2000; Ploughman et al., 2005; Chang et al., 2011) and can regulate microglial number, morphology, and mRNA profile (Falomir-Lockhart et al., 2019; Ivan et al., 2023). Exercise-induced increases in NGF have been observed in the hippocampus and cortex (Neeper et al., 1996; Molteni et al., 2002), and can regulate the microglial phenotype, including phagocytic function (Rizzi et al., 2018; Fodelianaki et al., 2019). Exercise-induced increases in neurotransmitters, including norepinephrine, dopamine, and serotonin, are widespread throughout the brain (Vecchio et al., 2018). Norepinephrine regulates microglial arborization and dynamics (Gyoneva and Traynelis, 2013; Liu et al., 2019; Stowell et al., 2019), and has been shown to increase with exercise in many brain areas, including the cerebellum, striatum, hypothalamus, midbrain, cortex, spinal cord, and pons/medulla (Brown et al., 1979; Semenova et al., 1981; Meeusen and De Meirleir, 1995; Pagliari and Peyrin, 1995; Dunn et al., 1996; Dishman et al., 2000). Serotonin levels increase in the hippocampus, midbrain, hypothalamus, striatum, and cortex with exercise (Bailey et al., 1993; Gomez-Merino et al., 2001), and serotonin can regulate microglial phagocytic activity (Krabbe et al., 2012) and directional motility (Kolodziejczak et al., 2015; Etienne et al., 2019). Additionally, exercise increases dopamine in the striatum, nucleus accumbens, midbrain, hypothalamus, and hippocampus (Chaouloff et al., 1987; MacRae et al., 1987; Mathes et al., 2010) and regulates the RAS activity and levels of angiotensin receptors in microglia, resulting in an anti-inflammatory effect (Dominguez-Meijide et al., 2017). Dopamine also regulates microglia migration (Färber et al., 2005; Mastroeni et al., 2009), phagocytosis (Fan et al., 2018), and morphology (Fan et al., 2018). In summary, there is compelling evidence that exercise could regulate microglia activity through one or many of these mechanisms. However, the translatability of these effects to human and how they pertain to changes in cognition remains to be determined. In humans, there is evidence showing exercise-related changes in BDNF are associated with improved executive performance (Hwang et al., 2016). However, a recent study found cognitive improvement following resistance and aerobic exercise was not associated with peripheral biomarkers including adrenaline, noradrenaline, glucose, lactate, cortisol, IGF-1, or BDNF in humans (Ando et al., 2022). Further research is needed to tie exercise-induced changes in central or peripheral biomarkers to altered microglia activity and enhanced cognitive performance.

## New frontiers for understanding how exercise regulates microglia

Numerous studies have explored how physical activity can impact microglia in various animal models, yet there are several understudied areas. Advanced *in vivo* imaging techniques allow scientists to track cellular structures over long periods of time, creating a wealth of opportunity to better understand how exercise regulates brain physiology. These methods have already been applied to track blood vessels (Cudmore et al., 2017) and dendritic spines (Chen et al., 2017) chronically over time in living mice undergoing different exercise regimens. However, knowledge regarding how exercise influences microglia dynamic functions is limited. Given the numerous neurotransmitters and neurotrophic factors that are known to regulate microglial activity (Figure 9), investigation of the effects of PE on microglia functions in other models is warranted. It is also possible that microglial interactions with specific components of their environment may also be changed by exercise. For example, microglia are known to make physical contacts with dendritic spines to facilitate structural plasticity and regulate neuronal health in different areas of the brain (Tremblay et al., 2010; Nebeling et al., 2023), and these interactions could be differentially regulated by physical activity. Employing reporter mice that label multiple brain structures or cells for chronic *in vivo* imaging could provide deeper insights into how exercise regulates microglial interactions with specific components of their environment over time.

Advancements in sequencing technologies also provide great potential for a comprehensive examination of microglia phenotypes. Several studies reviewed here used bulk tissue RNA sequencing to elucidate the mechanisms behind exercise effects on the brain (Wassouf et al., 2018; Wang J. et al., 2021; Yang et al., 2023). However, this methodology is limited in that it only provides average gene expression patterns across of population of heterogenous cells. However, with the emergence of single cell technologies, which allow researchers to examine changes in gene expression of individual cells, there is ample opportunity to examine microglial states in health and disease within the contexts described in this review (Ochocka and Kaminska, 2021). Indeed, a recent report by Sun et al., used single-cell transcriptomics in young and old mice exercising for 12 months to identify exercise effects across 14 different tissues (Sun et al., 2023), finding age-related changes in gene expression and increases in IBA1 expression were ameliorated by exercise in the cortex, dentate gyrus, cerebellum, and spinal cord. Intriguingly, the authors found that out all the tissues examined in the study, the aged central nervous system tissues were mostly strongly impacted by exercise, reinforcing the sentiment that there is high potential for exercise to benefit brain health (Sun et al., 2023). Overall, much remains to be discovered regarding the influence of physical exercise on microglial functions within and between brain regions in various diseases states.

## Conclusion

There is strong evidence that PE can be beneficial in many rodent models of disease. Despite this, there are several areas where more research on exercise-induced changes in microglial function could yield important insights, particularly in conjunction with other lifestyle factors, where regional- and sex-dependent responses to different exercise paradigms may be more nuanced. Additionally, utilizing PE as an intervention in neurodevelopmental disorders, while challenging, could prove to be effective. Lastly, using newer technologies that allow for *in vivo* tracking of microglia dynamics simultaneously with other cellular structures, and carefully phenotyping microglia on the transcriptomic level during PE could uncover mechanisms underlying the beneficial effects of exercise on cognition.

## Author contributions

AS: Conceptualization, Writing – original draft, Writing – review & editing. AM: Funding acquisition, Writing – review & editing.

## Figures and Tables

**Table:** 

	
PE	physical exercise
CNS	central nervous system
CD68	cluster of differentiation 68
CX3CR1	C-X3-C motif chemokine receptor 1
CD86	cluster of differentiation 86
MHCII	major histocompatibility complex class 2
IGF-1	insulin-like growth factor 1
BDNF	brain-derived neurotrophic factor
C1QA	complement C1q A Chain
CD206	mannose receptor
Gal-3	galectin-3
FASD	fetal alcohol spectrum disorders
mTOR	mammalian target of rapamycin
VWR	voluntary wheel running
CDLK5	cyclin-dependent kinase-like 5
Shank3	multiple ankyrin repeat domains 3
FMRP	fragile X messenger ribonucleoprotein 1 protein
Mecp2	X-linked methyl-CpG-binding protein 2
CR3	complement receptor 3
IL-1β	interleukin-1 beta
IL-18	interleukin 18
IL-6	interleukin 6
Iba1	ionized calcium-binding adapter molecule 1
NL3^R451C^	neuroligin-3
HD	Huntington’s disease
ALS	amyotrophic lateral sclerosis
AD	Alzheimer’s disease
MS	multiple sclerosis
PD	Parkinson’s disease
APP	amyloid protein precursor
PSEN1	presenilin-1
PSEN2	presenilin-2
MAPT	microtubule-associated protein tau
TREM2	triggering receptor expressed on myeloid cells 2
EAE	experimental autoimmune encephalomyeliti
MPTP	1-methyl-4-phenyl-1,2,3,6-tetrahydropyridine
TDP-43	TAR DNA binding protein 43 kDa
ICH	intracerebral hemorrhage
OX-6	major histocompatibility complex class 2 1a
TNF-α	tumor necrosis factor alpha
MSG	monosodium glutamate
Cox-2	cyclooxygenase 2
DEET	N, N-diethyl-m-toluamide
PER	insecticide permethrin
PB	pyridostigmine bromide
O3	ozone
Pb	lead
NLRP	nucleotide-binding oligomerization domain, leucine rich repeat and pyrin domain containing
NGF	nerve growth factor
	

## Supplementary material

Supplementary Figure 1 Comprehensive literature review process. Studies were reviewed from the PUBMED search query: ((((exercise[Title/Abstract]) OR (physical exercise[Title/Abstract])) OR (physical activity[Title/Abstract])) AND (microglia[Title/Abstract])) NOT (review[Publication Type]). Four research studies within the scope of this review which were found manually outside the search parameters stated above were also included. Data was manually extracted from each study on microglia parameters in various exercise animal models. Reasons for exclusion are as listed.

Supplementary Table 1 Information extracted from included studies.
