# Supplementary figures and images for: Physical exercise regulates microglia in health and disease
Source: Front Neurosci. 2024 Jun 7;18:1420322. doi: 10.3389/fnins.2024.1420322 (PMC11192042; doi:10.3389/fnins.2024.1420322)

# Comprehensive Literature Review

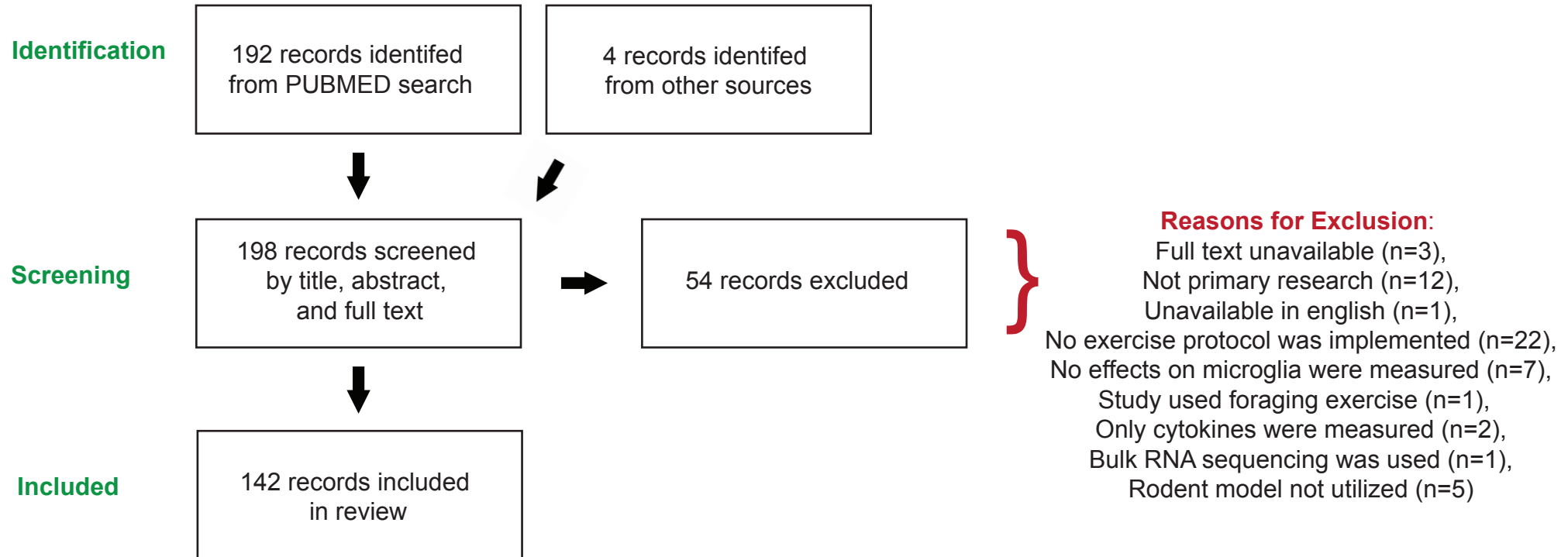

Supplement: Supplementary Figure 1 — Comprehensive literature review process. Studies were reviewed from the PUBMED search query: ((((exercise[Title/Abstract]) OR (physical exercise[Title/Abstract])) OR (physical activity[Title/Abstract])) AND (microglia[Title/Abstract])) NOT (review[Publication Type]). Four research studies within the scope of this review which were found manually outside the search parameters stated above were also included. Data was manually extracted from each study on microglia parameters in various exercise animal models. Reasons for exclusion are as listed. [file Data_Sheet_1.PDF]
